# Temporal Relationships Exist Between Cecum, Ileum, and Litter Bacterial Microbiomes in a Commercial Turkey Flock, and Subtherapeutic Penicillin Treatment Impacts Ileum Bacterial Community Establishment

**DOI:** 10.3389/fvets.2015.00056

**Published:** 2015-11-20

**Authors:** Jessica L. Danzeisen, Jonathan B. Clayton, Hu Huang, Dan Knights, Brian McComb, Shivdeep S. Hayer, Timothy J. Johnson

**Affiliations:** ^1^Department of Veterinary and Biomedical Sciences, University of Minnesota, Saint Paul, MN, USA; ^2^Department of Biomedical Informatics and Computational Biology, University of Minnesota, Minneapolis, MN, USA; ^3^Department of Computer Science and Engineering, University of Minnesota, Minneapolis, MN, USA; ^4^Biotechnology Institute, University of Minnesota, Saint Paul, MN, USA; ^5^Willmar Poultry Company, Willmar, MN, USA; ^6^Mid-Central Research and Outreach Center, University of Minnesota, Willmar, MN, USA

**Keywords:** poultry, avian, Turkey, bacteria, penicillin, microbiome, antibiotic, growth promoter

## Abstract

Gut health is paramount for commercial poultry production, and improved methods to assess gut health are critically needed to better understand how the avian gastrointestinal tract matures over time. One important aspect of gut health is the totality of bacterial populations inhabiting different sites of the avian gastrointestinal tract, and associations of these populations with the poultry farm environment, since these bacteria are thought to drive metabolism and prime the developing host immune system. In this study, a single flock of commercial turkeys was followed over the course of 12 weeks to examine bacterial microbiome inhabiting the ceca, ileum, and corresponding poultry litter. Furthermore, the effects of low-dose, growth-promoting penicillin treatment (50 g/ton) in feed on the ileum bacterial microbiome were also examined during the early brood period. The cecum and ileum bacterial communities of turkeys were distinct, yet shifted in parallel to one another over time during bird maturation. Corresponding poultry litter was also distinct yet more closely represented the ileal bacterial populations than cecal bacterial populations, and also changed parallel to ileum bacterial populations over time. Penicillin applied at low dose in feed significantly enhanced early weight gain in commercial poults, and this correlated with predictable shifts in the ileum bacterial populations in control versus treatment groups. Overall, this study identified the dynamics of the turkey gastrointestinal microbiome during development, correlations between bacterial populations in the gastrointestinal tract and the litter environment, and the impact of low-dose penicillin on modulation of bacterial communities in the ileum. Such modulations provide a target for alternatives to low-dose antibiotics.

## Introduction

Turkey meat is one of the leanest meat sources of protein available, and its production is a multibillion dollar per year U.S. industry (1). Nearly 250 million turkeys are grown each year, making the U.S. the world’s largest producer of turkeys and the biggest exporter of turkey products. The U.S. produces 7.5 billion pounds of turkey meat per year, and this number is steadily increasing.

The gastrointestinal health of an animal is key to its successful growth and development. For many years, gut health and development in U.S. commercial poultry has been routinely managed through the use of low-dose levels of antibiotics in feed to prevent diseases, improve overall flock consistency, and increase final body weights (2). Even with the use of low-dose antibiotics, gut health issues still occur. For example, turkey flocks are still plagued by a condition known as “Light Turkey Syndrome,” or LTS (3, 4). LTS has not been attributed to any known pathogen or management practice (3), yet some farms yield market weights 1–3 pounds below the national average, using the same source of poults as farms achieving these weight goals. Higher doses of some antibiotics can alleviate these problems, but they present their own set of problems related to the development of antibiotic resistant bacterial pathogens that threaten both human and animal health. With an ongoing movement to withdraw the use of low-dose antibiotics on poultry farms, alternatives to antibiotics are greatly needed to sustain health and performance in commercial turkey flocks.

In the avian intestinal bacterial community, it is well established that great differences exist from a spatial (proximal to distal) standpoint (5). The chicken ileum and ceca alone are thought to harbor at least 10^8^ and 10^11^ organisms per gram of digesta, respectively (6), and this density is achieved within days after hatch (6). However, the taxonomic composition of these microbes changes rapidly during the first week of development. Early studies involving denaturing gradient gel electrophoresis (DGGE) revealed that the chicken cecal microbiome, while quite diverse, is dominated by a small subset of conserved bacterial species in mature birds (7, 8). In general, the cecal microbiome is dominated by Clostridiales, and the small intestine is dominated by Lactobacillales (9). However, the avian microbiome is highly dependent on bird age, and there is great diversity at the bacterial species level (10). Most microbiome-based studies in both chickens and turkeys have focused on the microbiome related to carriage of pathogens (11–13). Because of a primary emphasis on pathogens in the more distal portions of the intestinal tract, fewer studies have examined the ileum microbiome. Pioneering work by Lu et al. examined the ileal and cecal bacterial communities of the chicken during bird development (14). They found that the broiler ileum was dominated by Lactobacillaceae, whereas the cecum was dominated by Clostridiaceae.

A number of studies have sought to examine the effects of antibiotic growth promoters (AGPs) on the intestinal microbiome. For example, virginiamycin and other AGPs applied in broilers were shown to exert the greatest modulatory effect on the proximal small intestinal microbiome correlating with increased average daily weight gain, as compared to the distal intestine and ceca (9, 15). Several AGPs [avilamycin, bacitracin methylene disalicylate (BMD), and enramycin] applied to broilers housed in floor pens resulted in improvements in growth performance, grossly correlated with changes to the intestinal microbiota (16). An interesting result validated through multiple studies is that AGP treatment decreased bacterial diversity in the avian ileum and decreased *Lactobacillus* populations (17–19). AGPs also appear to decrease bird-to-bird variations in weight and performance (20).

Penicillin G procaine has been shown to enhance weight gain and feed efficiency in commercial poultry (2). However, the underlying mechanisms by which administration of penicillin in feed is effective have not been fully examined. The purpose of this study was to define the baseline correlations between the bacterial populations inhabiting the turkey cecum, ileum, and surrounding litter environment and to assess the impact of penicillin in feed on the ileum bacterial microbiome of turkeys during the early brood period when the turkey gastrointestinal microbiome is most dynamic (10).

## Materials and Methods

### Study Design

All studies were performed on commercial turkeys; therefore, ethical standards for commercial turkey production were followed by the company performing the study. Animals were euthanized using methods approved by the American Veterinary Medical Association. Two trials were performed at a commercial turkey research facility in Willmar, Minnesota. The barn was divided into 24 pens, with 16 pens on one half of the barn each housing 1,500 turkey poults, and 8 pens on the other half of the barn each housing 3,000 birds. Feed was mixed and supplied using a Feedlogic robot (Feedlogic Corporation, Willmar, MN, USA). In the first trial, Hybrid Converter female poults (Willmar Poultry Company, Willmar, MN, USA) were placed at day-of-hatch into a pen housing 3,000 birds. At days 7, 14, 21, 28, 35, 42, 56, 70, and 84, five birds were randomly selected and euthanized. For this trial, birds were moved to a commercial grow-out facility at 5 weeks and were separated from other commercial birds by fencing within the grow-out barn. They were subsequently raised under standard commercial turkey management practices. The following measurements were taken from each bird sampled: total body weight, intestinal weight, intestinal length, and cecal score. Cecal score was recorded throughout the study by a single person blinded to the experimental groups. The scoring system ranged from 0 to 3, based upon consistency, color, and gas present in the cecal contents. A score of 0 indicated pasty and dark brown cecal content; a score of 1 indicated changes in consistency toward watery but still dark brown content; a score of 2 indicated changes in color and consistency of the cecal contents toward watery and yellow color; and a score of 3 indicated watery, gassy, and yellow-colored cecal content. Both ceca and the ileum from euthanized poults were aseptically collected intact, homogenized, and frozen at −20°C for future processing. Grab litter samples of representative bedding from each group were aseptically collected in whirl-pak bags at the same time points. Each litter sample collected was composed of a pool of five samples collected randomly from dry areas in the barn not including fecal or cecal droppings.

In the second trial, four pens were used, including two replicates each of control and treatment groups. Birds were immediately placed on standard feed containing 50 g/ton of BMD for control birds, and 50 g/ton BMD plus 50 g/ton penicillin G procaine for treatment birds. At days 7, 14, and 21, five birds per pen were randomly selected and euthanized. The following measurements were taken: total body weight, intestinal weight, intestinal length, and cecal score. Ilea from euthanized poults were aseptically collected intact, homogenized, and frozen at −20°C for future processing.

### Sample Processing and Sequencing

DNA was extracted using a bead-beating procedure and the QIAmp^®^ DNA Stool Kit (Qiagen, Valencia, CA, USA) as previously described (21). PCR was used to amplify the V3 hypervariable region of the 16S rRNA gene using primers containing Illumina barcoding and sequencing primers, as well as sample-specific barcodes on the reverse primers, as previously described (22). The PCR conditions used were an initial denaturation step at 95°C for 2 min, followed by 25 cycles of 95°C for 30 s, 60°C for 30 s, and 72°C for 30 s, with a final extension at 72°C for 7 min. The PCR product was excised from a 1.5% gel and purified using the QIAquick Gel Extraction Kit following manufacturer’s instructions (Qiagen). Sample DNA quality and quantity were assessed on a Bioanalyzer 2100 (Agilent, Palo Alto, CA, USA) using a DNA-1000 lab chip. Sequencing was performed at the University of Minnesota Genomics Center using Illumina MiSeq paired-end 2 × 250 bp technology.

### Data Analyses

Following sequencing, sorting by barcode was performed to generate fastq files for each sample. Paired-end reads were assembled and quality screened using Pandaseq, using a threshold quality cut-off value of 0.6 and eliminating any assembled reads with ambiguous base calls (23). Proximal and distal primers were trimmed from the sequence reads. Random subsets of 20,000 high-quality reads per sample for Trial #1 and 40,000 reads per sample for Trial #2 were selected using the sub.sample approach in Mothur (24). In total, 45 cecum samples, 45 ileum samples, and 16 pooled litter samples were analyzed from Trial #1 (2.12 million reads), and 30 ileum samples were analyzed from Trial #2 (1.2 million reads). A *de novo* operational taxonomic unit (OTU) picking approach was used in QIIME (25) using uclust (26) independently for each dataset (Trial #1 and Trial #2). OTUs containing <25 sequences were removed to eliminate possible spurious OTUs due to sequencing error. Potential chimeras were removed using ChimeraSlayer (27). QIIME was used for assessments of alpha diversity, beta diversity using Unifrac (28), and phylogenetic classifications using the RDP database (29). Differential abundances of OTUs and other phylogenetic classifications were identified using METASTATS (30). Construction of heatmaps was performed using the R statistical software (31). Statistical analyses for differences in community structure were performed using distance matrices analyzed via the AMOVA command in Mothur (24). A paired two-sample *t*-test was used to statistically compare total body weights, intestinal weights, intestinal lengths, and cecal scores at each time point during trial #2.

The data from this project is freely available at the Data Repository for the University of Minnesota (DRUM) at the following link: http://hdl.handle.net/11299/174930.

## Results

### Trial #1: Relationships Between Cecum, Ileum, and Litter Bacterial Microbiomes During Flock Succession

**Figure 1:**
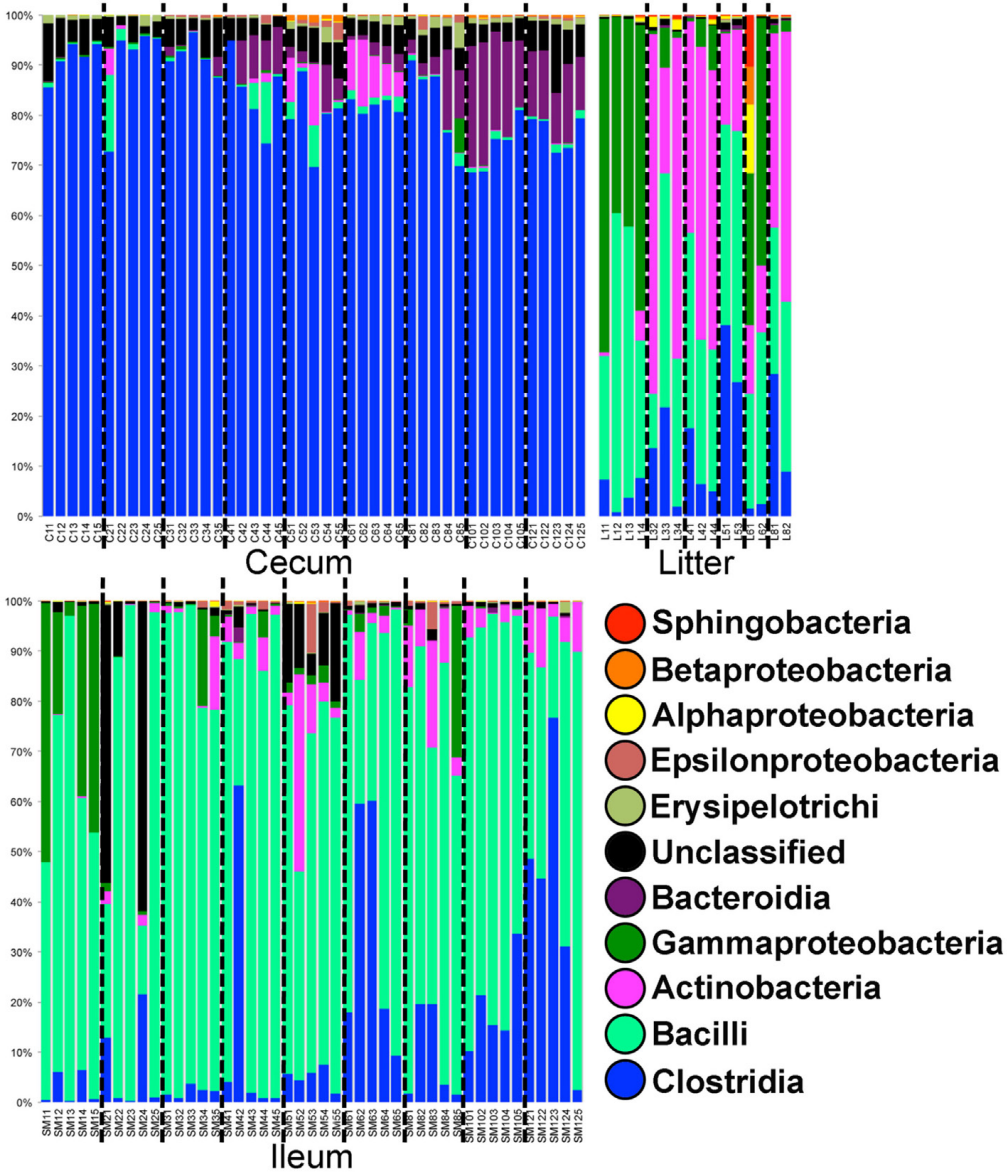
**Class-level taxonomic compositions of bacterial microbiome samples in Trial #1**. For each sample type (ileum = SM, litter = L, and cecum = C) individual samples are depicted by age (weeks 1–12) followed by bird number (1–5). Black dashed lines divide samples by age.

In Trial #1, 38,861 OTUs were identified using open reference OTU picking. Our goal was to assess the dominant bacterial populations and avoid spurious OTU calling due to sequencing error. After removing OTUs with <25 total sequencing reads, 1,101 OTUs remained in the dataset. Using the greengenes database, OTUs were classified at the bacterial class level (Figure 1). In cecal samples, Clostridia was the dominant class (>70% of the population) throughout trial 1 with lower levels of Bacteroidia (5–20% of the population) appearing at 28 days and beyond. Levels of Gammaproteobacteria remained low throughout, typically <1% of the total population. In contrast, Bacilli was the dominant bacterial class in ileum samples throughout trial 1 (50–90% of the population), with levels of Clostridia increasing (0–75% of the population) throughout the study. Gammaproteobacteria were high (up to 50% of the population) in ileum samples at day 7, then decreased substantially in the subsequent weeks. Actinobacteria also increased in abundance in ileum samples (5–30% of the population) at day 28 and beyond. Litter samples were distinct from both ceca and ileum in terms of bacterial class composition, but more closely represented ileum samples than ceca samples. Notably, Gammaproteobacteria and Actinobacteria were of substantially higher relative abundance in litter samples at day 7 and days 21–56, respectively.

**Figure 2:**
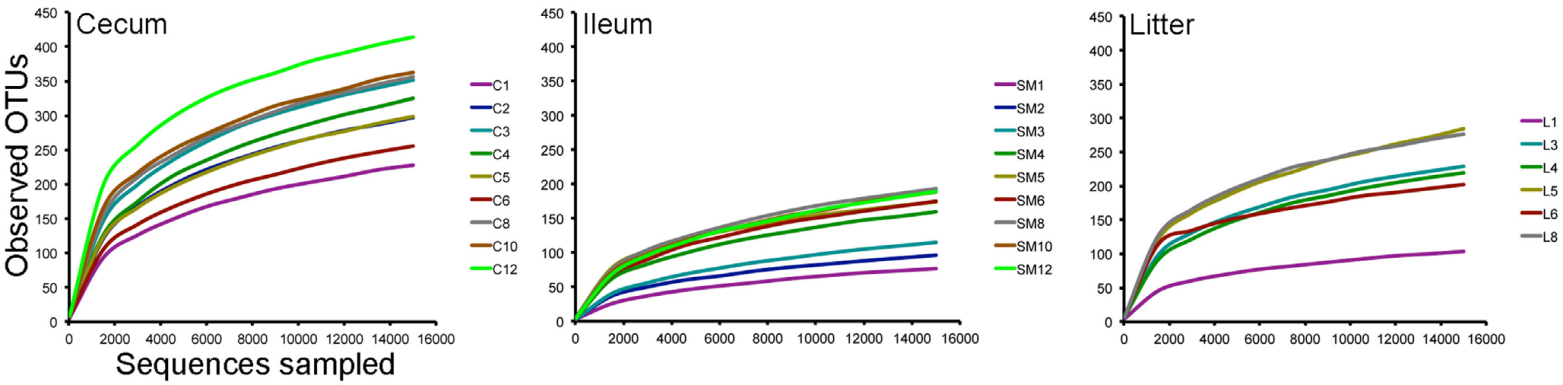
**Rarefaction curves of cecum, ileum, and litter samples**. Legends refer to sample type (C = cecum, SM = ileum, L = litter) and weeks of age (1–12) for each sample type.

Using an OTU-based approach, bacterial species richness was highest in ceca samples throughout the study, followed by litter and ileum samples (Figure 2). Species richness increased in samples through 35 days, when the birds were moved to a commercial grow-out barn. At day 42, following movement to the grow-out barn and change in feed to reduced protein composition, bacterial species richness decreased in the ceca and litter samples but remained the same in ileum samples. At day 56, bacterial species richness in these samples increased to pre-movement levels or greater. Ileum and ceca samples continued to increase in species richness through day 84. Using a non-parametric two-sample *t*-test with Bonferroni correction, alpha diversity measurements were significantly different (*P* = 0.003) comparing ileum versus cecum samples (*P* = 0.003) and litter versus cecum samples (*P* = 0.003), but not ileum versus litter samples.

**Figure 3:**
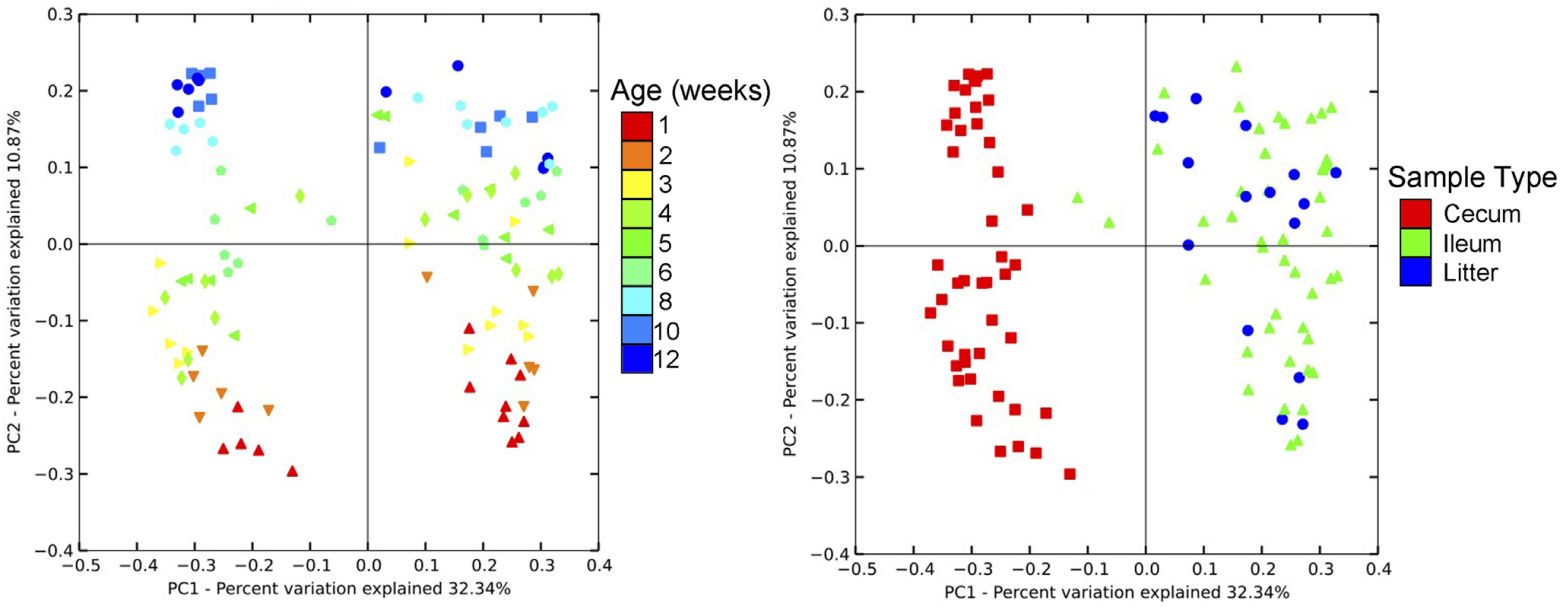
**Principal coordinates analysis (PCoA) of individual samples from turkey cecum, ileum, and litter within a single flock**. Left image depicts PCoA by flock age, and right image depicts PCoA by sample type.

Community-level similarities in the bacterial microbiome of samples were compared using principal coordinates analysis (PCoA; Figure 3). Samples were stratified primarily by age of flock, and also separated by sample type. Using AMOVA based upon distance matrix, bacterial communities from all three sample types were distinct (*P* < 0.001). Upon visualization of the PCoA plots, litter and ileum samples had some overlap, while both of these sample types were clearly distinct from cecal samples. However, all sample types shifted similarly over time on the plot, indicating that bird age has a predominant impact on the barn bacterial microbiome irrespective of sample type.

**Figure 4:**
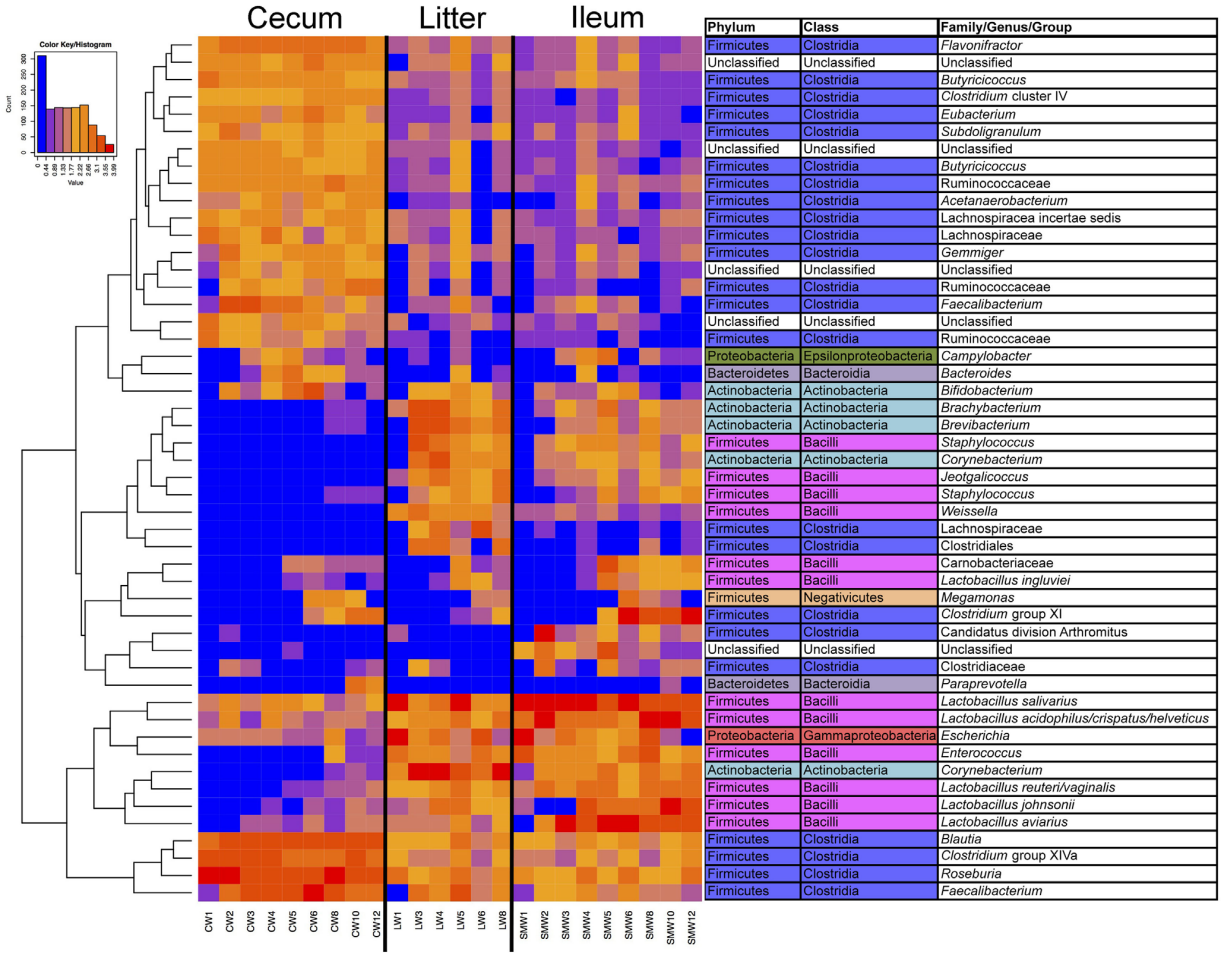
**Heatmap depicting abundance of the top 50 OTUs by overall abundance across samples, averaged by age/sample**. Heatmap was constructed using normalized log_10_ abundance of each OTU in each sample type. To the right of the heatmap is a table depicting classification of each OTU using RDP database assignment or best-hit classification where appropriate. “W” depicts the age of sample in weeks.

Samples were also analyzed at the OTU level for specific subsets of OTUs representing sample types and age (Figure S1 in Supplementary Material; Figure 4). It was clear from this analysis that there were shared subsets of OTUs present across all samples, unique subsets that were defining of a particular sample type(s), and OTUs that were dependent on flock age. For example, numerous OTUs were identified belonging to the phylum Firmicutes and class Clostridia that were dominant in cecal samples, and present in ileum and litter samples but at much lower abundance. Similarly, OTUs belonging to phylum Firmicutes and class Bacilli were dominant in ileum and litter samples but present in cecal samples at much lower abundance. OTUs classified at the genus level such as *Brachybacterium*, *Brevibacterium*, *Staphylococcus*, *Corynebacterium*, *Jeotgalicoccus*, and *Weissella* were found in ileum and litter samples, but were absent from cecal samples. An OTU that we had previously identified as Candidatus division Arthromitus (10) was identified in ileum samples but not found in litter or cecal samples. Finally, some OTUs displayed a temporal trend and were found more prominently in later-aged samples, such as those classified as *Lactobacillus aviarius*, *Lactobacillus johnsonii*, Clostridium group XI, *Megamonas*, and *Lactobacillus ingluviei*. OTU-based clustering of the sample confirmed what was observed with PCoA-based analysis, with cecum samples clearly separating from ileum/litter samples which contained considerable overlap in bacterial microbiome composition (Figure S2 in Supplementary Material).

### Trial #2: Effects of Low-Dose Penicillin Treatment on the Ileum Bacterial Microbiome

The effect of low-dose penicillin (50 g/ton) in feed on commercial turkey poults was examined in a second trial. Total bird weights were significantly higher at days 14 and 21 in the penicillin-treated groups compared to control groups (Table 1; *P* < 0.05). Intestinal weights and lengths were also significantly higher in penicillin-treated groups at day 21 (*P* < 0.05), but cecal score was unaffected (Table 2).

**Table 1:** **Total body weights of turkeys treated with and without 50 g/ton of penicillin in feed**.

	Day 7 weight (g)		Day 14 weight (g)		Day 21 weight (g)	
	Control	Penicillin	Control	Penicillin	Control	Penicillin
Average weight replicate 1	148.4	156.4	338	390.8^a^	665.8	779.8^a^
Average weight replicate 2	141.6	152.4	302.6	378.4^a^	664.4	736.8^a^
Standard deviation overall	21.6	18.0	54.1	44.5	111.2	59.4

*^a^Significantly different from control group (*P* < 0.05) using Student’s *t*-test*.

**Table 2:** **Intestinal measurements and cecal scores of turkeys with and without 50 g/ton of penicillin in feed**.

		Cecal score	Intestinal length (cm)	Intestinal weight (g)
Day 7	Control	1.5 ± 0.8	93.0 ± 6.4	18.2 ± 2.2
	Penicillin	1.5 ± 0.4	95.1 ± 6.4	16.5 ± 2.1
Day 14	Control	2.4 ± 0.5	122.3 ± 9.9	35.2 ± 5.5
	Penicillin	2.0 ± 0.7	127.4 ± 6.2	37.1 ± 3.7
Day 21	Control	1.6 ± 0.6	148.1 ± 7.4	52.0 ± 6.4
	Penicillin	1.8 ± 0.5	158.8 ± 10.8^a^	65.9 ± 11.9^a^

*^a^Significantly different from control group (*P* < 0.05) using Student’s *t*-test*.

**Figure 5:**
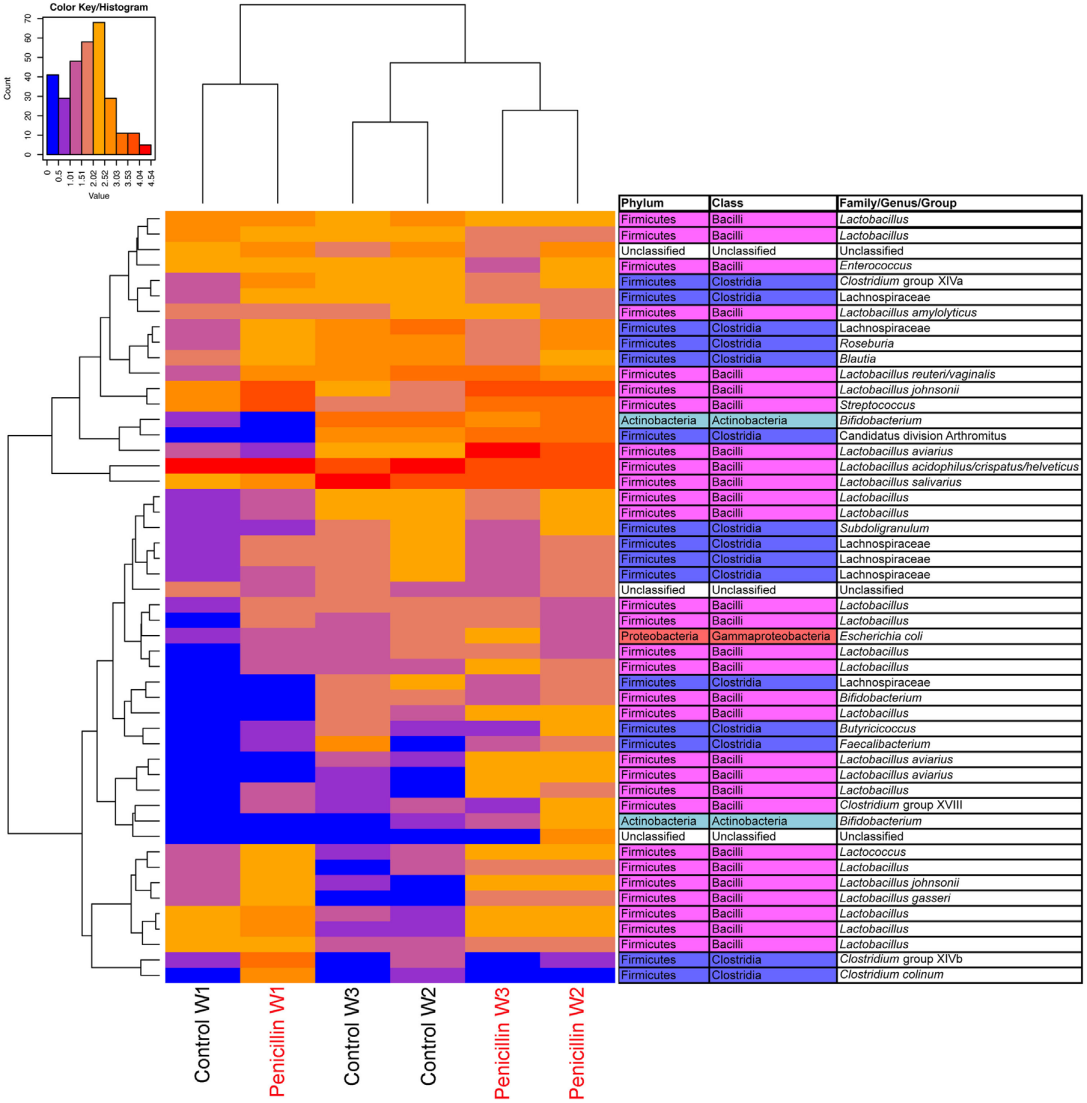
**Heatmap depicting abundance of the top 50 OTUs by overall abundance across samples, averaged by age/sample**. Heatmap was constructed using normalized log_10_ abundance of each OTU in each sample type. To the right of the heatmap is a table depicting classification of each OTU using RDP database assignment or best-hit classification where appropriate. “W” depicts the age of sample in weeks. Control = birds receiving standard feed with BMD, penicillin = birds receiving BMD plus penicillin in feed.

**Figure 6:**
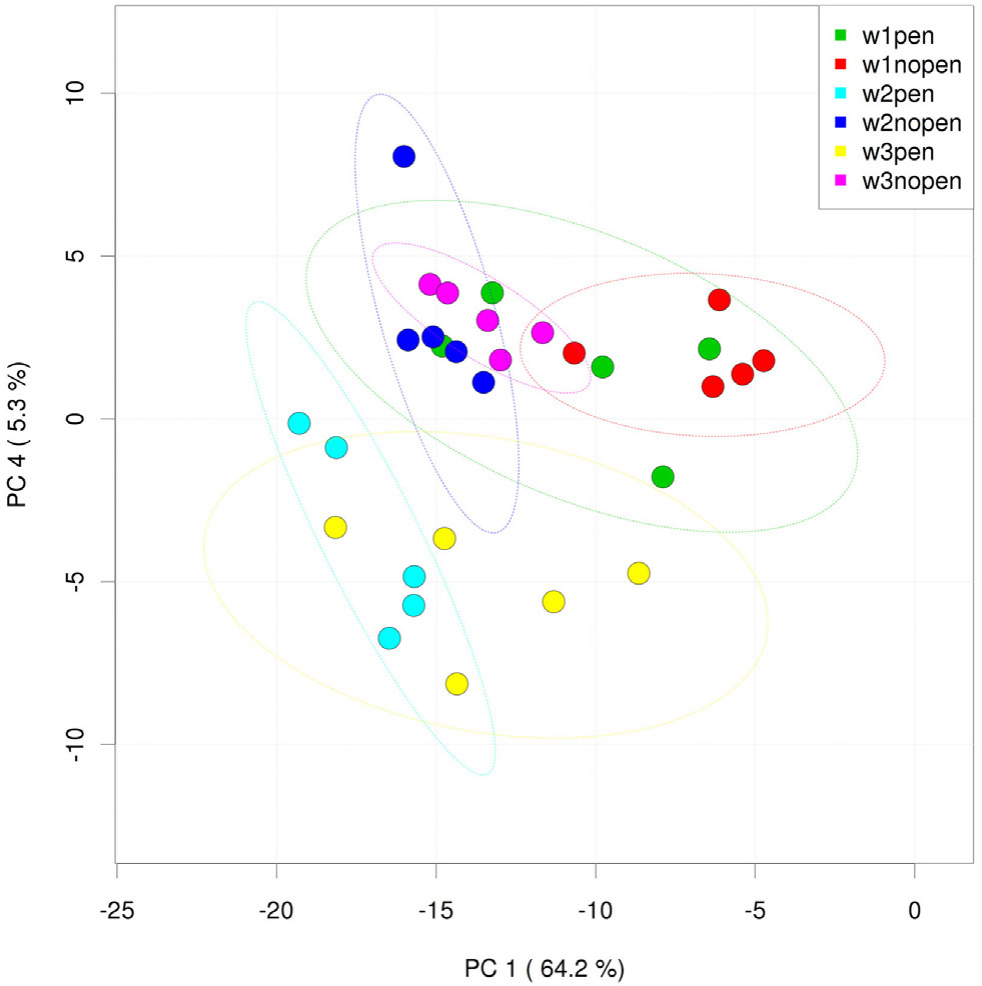
**Principal coordinates analysis (PCoA) of individual ileum samples from penicillin-treated versus control groups**. “W” = flock age in weeks; “pen” = penicillin-treated samples; “nopen” = control samples.

Analysis of the ileum bacterial microbiome was performed on five individual birds per treatment group and time point to determine if there were shifts in the microbiome associated with penicillin treatment. In total, 7,857 OTUs were identified from these samples using open reference OTU picking. After removing OTUs with <25 total sequencing reads, 786 OTUs remained in the dataset. OTUs were examined by treatment group and flock age (Figure 5), and hierarchical clustering suggested that penicillin treatment had effects on the ileum bacterial microbiome at weeks 2 and 3. AMOVA also revealed significantly different community structures in the control versus penicillin-treated groups at days 14 (*P* = 0.003) and 21 (*P* < 0.001). Using METASTATS comparison, OTUs that were of significantly higher abundance (*P* < 0.05) at days 14 and 21 in penicillin-treated groups included those classified as *L. aviarius*, *L. johnsonii*, *Streptococcus* sp., and several other *Lactobacillus* spp. that were unclassified beyond genus level (Data Sheet S1 in Supplementary Material). A PCoA plot confirmed bacterial community differences between control and treatment groups, with day 14 and day 21 samples shifted on the plot in the penicillin-treated groups (Figure 6). Shannon diversity and species richness were also assessed, and at days 7 and 14, the penicillin-treated groups were significantly increased compared to the control groups (*P* < 0.05) (Figure S3 in Supplementary Material).

## Discussion

Recent bans on the use of AGPs in the European Union (EU) broiler industry have been associated with numerous production-associated problems, including decreased feed efficiency, watery feces, and disease, among other conditions (32). Considering this, and that supplementation of antibiotics in poultry feed is a highly controversial issue (33), it is apparent that alternatives with similar modes of action are greatly needed. In order to identify these alternatives, we need to also understand the mechanisms by which antibiotic usage results in accelerated weight gain.

The purpose of Trial 1 was to examine the relationship between bacterial populations in the ileum, cecum, and litter within one flock over time. Age was a key factor in population shifts across all sample types, which has also been previously observed in turkey cecum and ileum studies (10, 21). Several OTUs were found predominantly in later time points across samples, including several classified *Lactobacillus* spp (*L. aviarius*, *L. johnsonii*, and *L. ingluviei*), which have been previously identified as key marker species of turkey ileum microbiome succession (10). In addition, *Clostridium* group XI was another OTU increasing in abundance with age of the flock; this diverse group includes *Eubacterium* and *Peptostreptococcus* spp, as well as *Clostridium bartlettii* (34), which may be another indicator of gut microbiome succession (10), in part due to the high ability of this species to ferment aromatic amino acids in the gut (35).

Sample type appeared to be another dominant factor affecting bacterial populations, with clear distinctions between the ileum and cecum populations; the litter samples were also distinct, but more closely represented the ileum. The differences between the cecum samples and ileum and litter samples were largely within the Firmicutes phylum; more specifically, Clostridia OTUs were enriched in the cecal samples, whereas Bacilli were more abundant in the ileum and litter. Several identified OTUs were present only in ileum and litter samples, including *Brachybacterium*, which was first detected in poultry litter (36), and *Brevibacterium*, a soil bacterium that has been used as an indicator organism for poultry waste contamination in the environment (37). Other OTUs identified only in the ileum and litter were *Staphylococcus*, *Corynebacterium*, *Jeotgalicoccus*, the latter of which is a lactic acid bacterium also found in the air in poultry houses (38), and *Weissella*, a genus reported in high abundance in healthy birds (39). A recently published study found that cecal content populations reflect cecal drop, whereas fecal drop populations were dissimilar to cecal populations, further suggesting that ileum populations would be more closely related to litter than to cecal community structure (40). However, another study found that the fecal microbiome represents a large portion of the cecal diversity, though it was not a good quantitative measure (41). The birds in the present study were placed on clean litter for the brood period (first 5 weeks), suggesting that the ileum microbiome influenced the litter microbiome; however, before week 6 the birds were moved into a commercial finisher barn setting, in which the bedding was mostly reused with just a thin layer of clean litter, which could have resulted in the litter microbiome influencing ileum populations from weeks 6 to 12. The reasons for litter more closely reflecting ileum are likely due to less frequent cecal discharge and a litter growth environment that would better support facultative anaerobes compared to strict anaerobes.

One OTU that was found only in the ileum samples was classified as Candidatus division Arthromitus, a segmented filamentous bacteria (SFB) previously reported to play a potential role in gut health in turkeys and other animals (10, 42). Little is known about this bacterium, as it has only recently been sequenced (43) and grown *in vitro* (44). SFBs are thought to be host-specific as they have reduced genomes and rely heavily on host metabolic functions, and very little is known about the turkey-specific strains; however, these bacteria have been reported to play a role in early innate immune system development in mice as well (45). Overall, while temporal succession of bacterial populations was observed in this flock similar to previous studies (10), differences in sample type were more prominent. In addition, our results corroborate findings that poultry fecal droppings or litter samples are a better predictor of ileal rather than cecal bacterial composition (40).

It is a well-known fact that antibiotics are commonly used as a feed additive in poultry operations as a way to enhance the growth of birds to reach market weights faster (46). In previous studies, supplementation of subtherapeutic levels of several antibiotics, such as penicillin, to poultry broiler feeds have been associated with increases in weight gain (9). Through our study, we have also shown that antibiotic usage, specifically penicillin combined with BMD, results in significantly increased weight gain in turkey broilers up to at least 3 weeks. A limitation of the study was that BMD was used in the control groups, thus the sole effects of penicillin on the microbiome were not identified in this study. However, the scenario used in these experiments reflects commonly applied practices in the turkey industry, so it is more relevant as a real-life application.

While limited information currently exists, hypotheses aimed at explaining mode of action of AGPs have been proposed, including shifts in microbiome composition in gastrointestinal tract act to improve feed efficiency (32). In our study, the relative increase in weight gain in penicillin-supplemented broilers as compared to control groups can be temporally correlated with shifts in microbiome composition. Given our findings, we believe mining the microbiome is a means for finding potential replacements for AGPs, such as by identifying specific bacterial taxa responsible for improving feed efficiency, which can be used as probiotics.

Of the probiotics that have been investigated as growth promoters in poultry, many have included *Lactobacillus* spp. (47). In our study, relative abundances of *Lactobacillus* spp., including *L. johnsonii* and *L. aviarius*, were higher in penicillin-supplemented groups as compared to control groups. In terms of growth promotion, *Lactobacillus* spp. have been associated with both beneficial and detrimental effects. One mechanism whereby lactobacilli have been shown to decrease weight gain in a pig model is through the production of bile salt hydrolase (BSH) (48). On the contrary, *Lactobacillus* spp. has been demonstrated in broiler chicks to antagonize pathogenic bacteria, thus resulting in weight gain (47). *Lactobacillus* spp., specifically *L. johnsonii*, has been shown to possess antibacterial activity against pathogenic bacteria (49), and Zulkifli et al. showed that feeding lactobacilli cultures to broiler chicks resulted in increased weight gain (50), comparable to feeding oxytetracycline. It seems plausible that supplementing poultry feeds with appropriate lactobacilli cultures could serve as an alternative method for improving feed efficiency in poultry flocks.

Few studies have investigated the ileum bacterial community structure focusing on commercial turkeys. Our data suggest that low-dose penicillin treatment has a discernable impact on ileum bacterial community structure. The initial effects during the first week of age increase bacterial diversity in the ileum, and subsequent effects apparently drive the ileal microbiome composition toward a state correlating with significant enhancements of body weight (10). Some important factors remain to be examined, such as the effects of AGP treatment on total microbial biomass and on the turkey immune system. Since penicillin treatment modulates the turkey ileal microbiome in a fashion similar to that previously observed between commercial turkey flocks with differing weight outcomes, modulating the ileal microbiome similarly using antibiotic-free approaches may provide an alternative approach by which to enhance performance and prevent disease in the commercial bird.

## Author Contributions

TJ and BM conceived the study. JD and BM performed the experiments. JC, HH, DK, and TJ analyzed the data. TJ, JC, JD, and SH wrote the manuscript.

## Conflict of Interest Statement

Dr. Brian McComb was an employee at Willmar Poultry Company at the time of this study. The remaining authors have no conflict of interest to declare.

## Supplementary Material

The Supplementary Material for this article can be found online at http://journal.frontiersin.org/article/10.3389/fvets.2015. 00056
