# Supplementary figures and images for: Temporal Relationships Exist Between Cecum, Ileum, and Litter Bacterial Microbiomes in a Commercial Turkey Flock, and Subtherapeutic Penicillin Treatment Impacts Ileum Bacterial Community Establishment
Source: Front Vet Sci. 2015 Nov 20;2:56. doi: 10.3389/fvets.2015.00056 (PMC4672264; doi:10.3389/fvets.2015.00056)

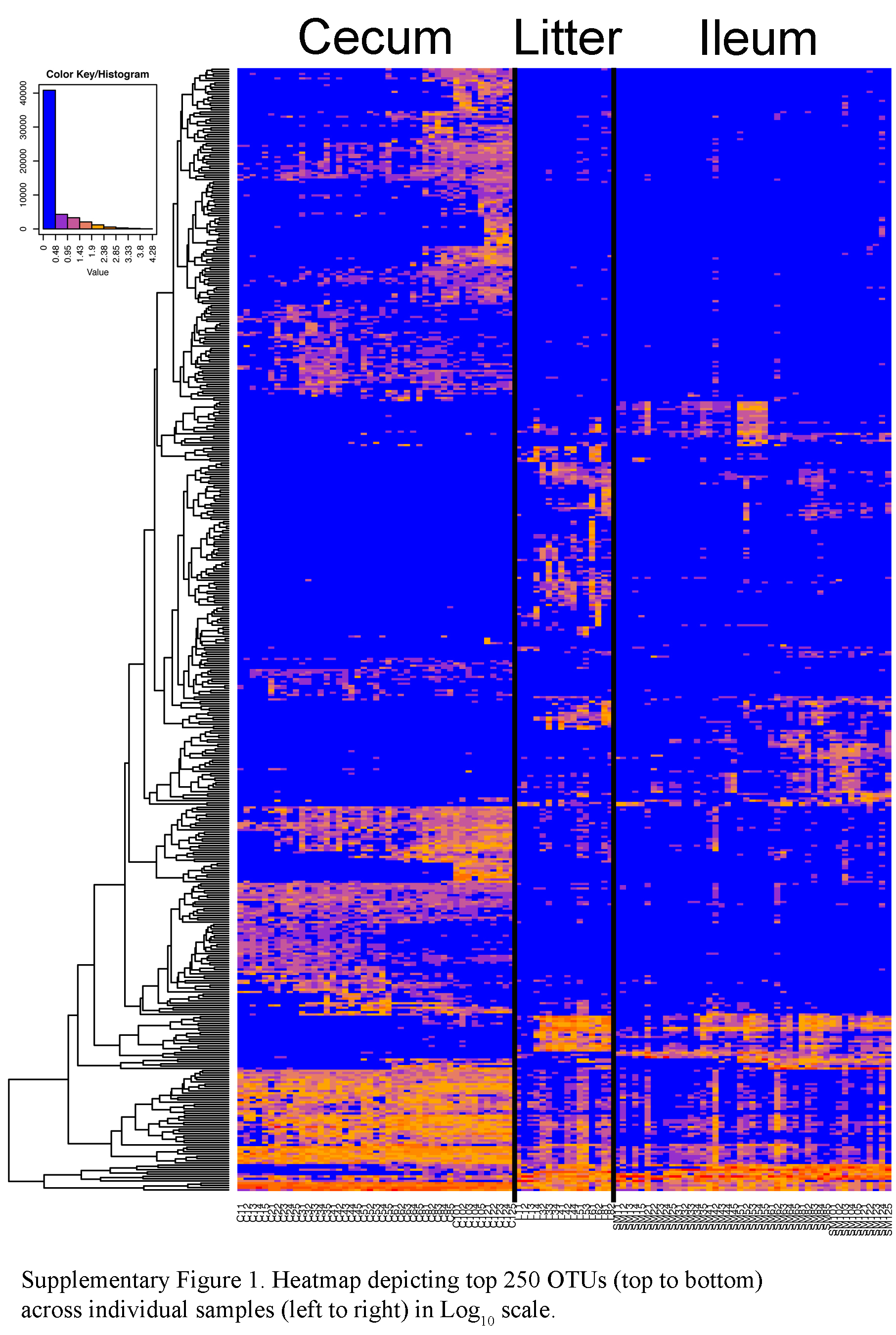

Supplement: Supplementary file 2 [file Image_1.JPEG]

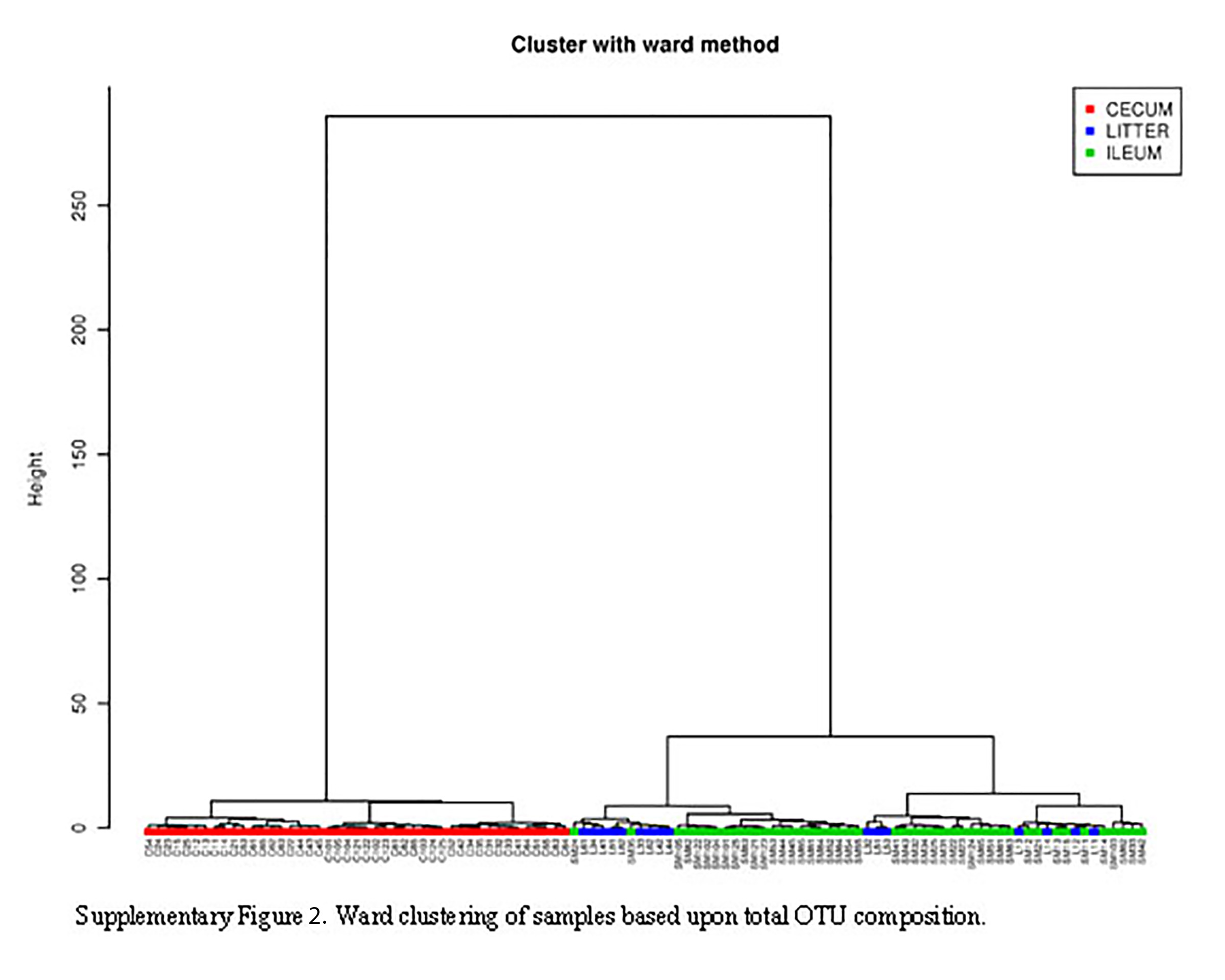

Supplement: Supplementary file 3 [file Image_2.JPEG]

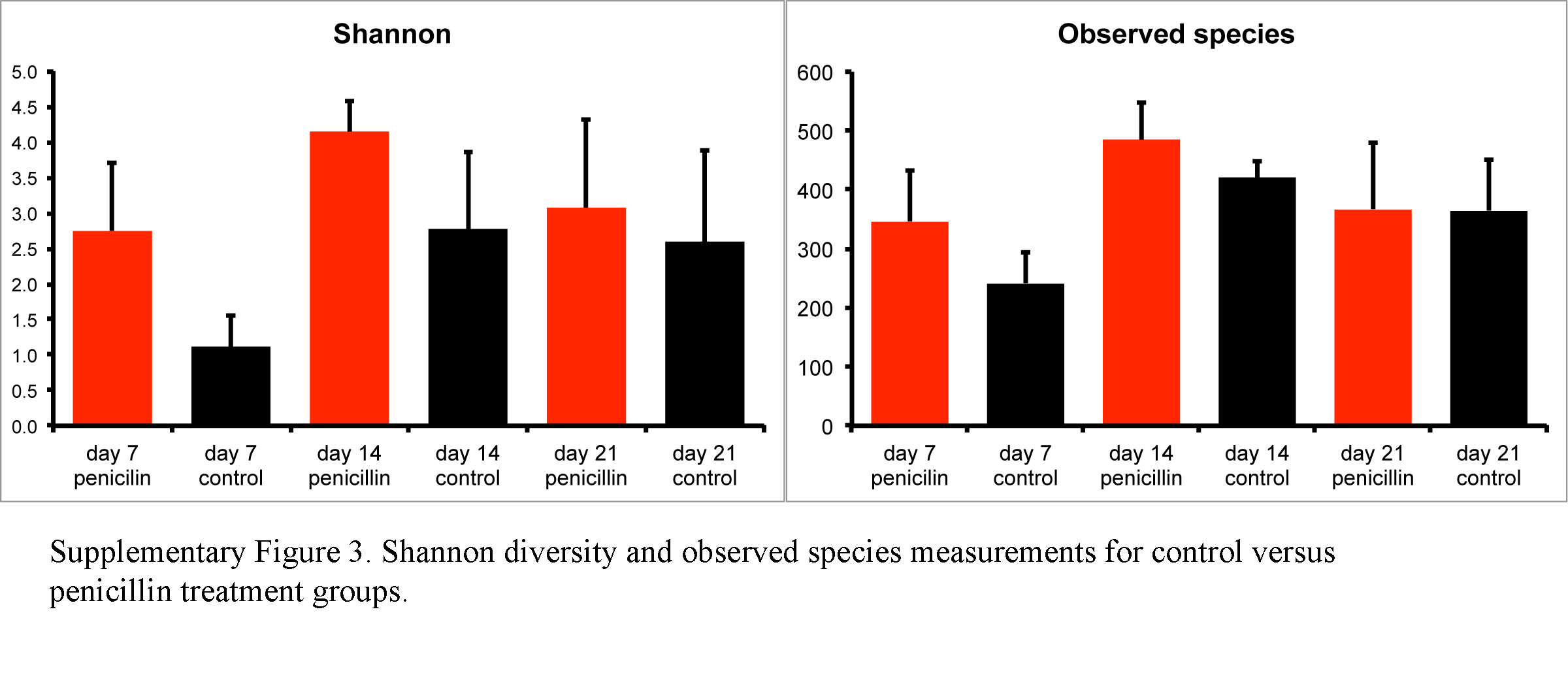

Supplement: Supplementary file 4 [file Image_3.JPEG]
